# Supplementary material for: Diagnostic accuracy of antigen detection in urine and molecular assays testing in different clinical samples for the diagnosis of progressive disseminated histoplasmosis in patients living with HIV/AIDS: A prospective multicenter study in Mexico
Source: PLoS Negl Trop Dis. 2021 Mar 8;15(3):e0009215. doi: 10.1371/journal.pntd.0009215 (PMC7971897; doi:10.1371/journal.pntd.0009215)
Supplement: S1 STARD Checklist — (PDF) [file pntd.0009215.s001.pdf]

| Section & Topic | No | Item | Reported on page # |
|---|---|---|---|
| **TITLE OR ABSTRACT** | | | |
| | **1** | Identification as a study of diagnostic accuracy using at least one measure of accuracy (such as sensitivity, specificity, predictive values, or AUC) | Title page 1.<br>Diagnostic accuracy of antigen detection in urine and molecular assays testing in different clinical samples for the diagnosis of progressive disseminated histoplasmosis in patients living with HIV/AIDS: A prospective multicenter study in Mexico. |
| **ABSTRACT** | | | |
| | **2** | Structured summary of study design, methods, results, and conclusions<br>(for specific guidance, see STARD for Abstracts) | Abstract; paragraph 1,2,3,4. |
| **INTRODUCTION** | | | |
| | **3** | Scientific and clinical background, including the intended use and clinical role of the index test | Introduction; paragraph 2,3,4<br>Overall, the clinical diagnosis of histoplasmosis represents a challenge. |
| | **4** | Study objectives and hypotheses | Introduction; paragraph 5.<br>Therefore, the purpose of this study was to determine the diagnostic accuracy of detecting *Histoplasma* antigen in urine using the IMMY ALPHA Histoplasma EIA kit (IAHE), IMMY ASR GM EIA kit (IAGE) and MiraVista lateral flow assays (MVLFA), as well as the Hcp100 and $1281\text{-}1283_{220}$ SCAR markers that use nested PCRs for diagnosing PDH among PLWHA compared to the definition of proven-PDH |
| **METHODS** | | | |
| *Study design* | **5** | Whether data collection was planned before the index test and reference standard<br>were performed (prospective study) or after (retrospective study) | Methods; paragraph 1<br>We conducted a multicenter, prospective, double-blinded, diagnostic test study. |
| *Participants* | **6** | Eligibility criteria | Methods; paragraph 1.<br>Men and women older than 18 years of age, with prior diagnosis of HIV infection and “suspected disseminated histoplasmosis” |
| | **7** | On what basis potentially eligible participants were identified (such as symptoms, results from previous tests, inclusion in registry) | Methods; paragraph 2.<br>Suspected PDH: any patient with any three of the following: fever, weight loss (>5% of total body weight in the last 6 months), lymphadenopathy, hepatomegaly, splenomegaly, mucous ulcers, skin lesions, gastrointestinal bleeding, diarrhea; bicytopenia or pancytopenia (hemoglobin <10 g/dL, total neutrophils count $<1.8\ x10^3/\mu L$, platelets $<100{,}000/\mu L$), elevation of two times over the normal upper limit of aspartate aminotransferase, lactic dehydrogenase and/or ferritin; radiographic evidence of extrapulmonary involvement (lymphadenopathy, hepatomegaly, splenomegaly, liver and/or spleen abscess, pancreatitis, cholecystitis, epididymitis, gastrointestinal, adrenal, brain, heart, bone, prostate or joint lesions) |
| | **8** | Where and when potentially eligible participants were identified (setting, location and dates) | Methods; paragraph 1<br>December 2015 to April 2018; ten reference centers distributed in seven states of Mexico participated (eight tertiary care centers and two secondary care general hospitals). |
| | **9** | Whether participants formed a consecutive, random or convenience series | Methods; paragraph 1<br>Men and women older than 18 years of age, with prior diagnosis of HIV infection and “suspected disseminated histoplasmosis” were consecutively identified |
| *Test methods* | **10a** | Index test, in sufficient detail to allow replication | Methods; paragraph 5,6,7,8,9,10,11,12 |
| | **10b** | Reference standard, in sufficient detail to allow replication | Methods; paragraph 3 |
| | **11** | Rationale for choosing the reference standard (if alternatives exist) | NA |
| | **12a** | Definition of and rationale for test positivity cut-offs or | Methods; paragraph 5,6,7,10 |

CC BY SA

| | | | |
|---|---|---|---|
| | | result categories<br>of the index test, distinguishing pre-specified from exploratory | |
| | **12b** | Definition of and rationale for test positivity cut-offs or result categories of the reference standard, distinguishing pre-specified from exploratory | Methods; paragraph 2<br>All cultures were processed as previously described.[17] |
| | **13a** | Whether clinical information and reference standard results were available to the performers/readers of the index test | Methods; paragraph 4<br>Preliminary and definitive results of the cultures and histopathological studies were reported to the patient's physician as soon as they were available to follow up the patients and define the treatment. |
| | **13b** | Whether clinical information and index test results were available to the assessors of the reference standard | Methods; paragraph 4<br>The results of the molecular and antigen detection tests were not informed, nor were they given any clinical value and were only of the knowledge of the principal investigator of this study. |
| *Analysis* | **14** | Methods for estimating or comparing measures of diagnostic accuracy | Methods; paragraph 13<br>We calculated the sensitivity, specificity, positive predictive value (PPV), negative predictive value (NPV), positive and negative likelihood ratios, with their respective 95% confidence intervals (95% CI) of the antigen urine tests and molecular tests, using as gold standard the cases classified as proven-PDH by culture and/or histopathology. The Kappa index and its respective 95% CI were used to determine the agreement between urine *Histoplasma* antigen detection tests. We use STATA 11.0 software (StataCorp LLC, College Station, TX) to perform the statistical analysis. |
| | **15** | How indeterminate index test or reference standard results were handled | Methods; Fig. 2, 4, and 5. |
| | **16** | How missing data on the index test and reference standard were handled | Methods; paragraph 1<br>For inclusion, at least a blood or a bone marrow culture in media supporting *H. capsulatum* growth and 20 ml of urine were required; according to the on-site physician´s criteria, additional tissue samples from diverse anatomical sites were obtained for culture and histopathology. Patients with incomplete clinical information and those who refused to participate in the study were excluded. |
| | **17** | Any analyses of variability in diagnostic accuracy, distinguishing pre-specified from exploratory | Methods; paragraph 13<br>We calculated the sensitivity, specificity, positive predictive value (PPV), negative predictive value (NPV), positive and negative likelihood ratios, with their respective 95% confidence intervals (95% CI) of the antigen urine tests and molecular tests, using as gold standard the cases classified as proven-PDH by culture and/or histopathology. The Kappa index and its respective 95% CI were used to determine the agreement between urine *Histoplasma* antigen detection tests. We use STATA 11.0 software (StataCorp LLC, College Station, TX) to perform the statistical analysis. |
| | **18** | Intended sample size and how it was determined | NA |
| **RESULTS** | | | |
| *Participants* | **19** | Flow of participants, using a diagram | Results; Fig 1 |
| | **20** | Baseline demographic and clinical characteristics of participants | Results; table 1 |
| | **21a** | Distribution of severity of disease in those with the target condition | Results; table 2 |
| | **21b** | Distribution of alternative diagnoses in those without the target condition | Results; paragraph 1.<br>Among the 280 patients without PDH, we found 57 (20%) mycobacterial infections (28 with *Mycobacterium tuberculosis*, 28 with *Mycobacterium avium* complex, and one case with *Mycobacterium tuberculosis* and *Mycobacterium avium* complex infection), 35 (13%) with other diagnosis (21 with bacteremia / fungemia [blood and/or bone marrow positive cultures] and 14 with histopathology diagnosis of CMV, Kaposi sarcoma, |

cc BY SA

| | | | |
|---|---|---|---|
| | | | lymphoma and/or HIV lymphadenopathy) and in 188 (67%) the diagnosis was not established. |
| | **22** | Time interval and any clinical interventions between index test and reference standard | NA |
| *Test results* | **23** | Cross tabulation of the index test results (or their distribution) by the results of the reference standard | Results; Fig 2, 4, 5. |
| | **24** | Estimates of diagnostic accuracy and their precision (such as 95% confidence intervals) | Results; table 3. |
| | **25** | Any adverse events from performing the index test or the reference standard | NA |
| **DISCUSSION** | | | |
| | **26** | Study limitations, including sources of potential bias, statistical uncertainty, and generalizability | Discussion; paragraph 12.<br>Our study has some limitations, we did not evaluate the possible interference of foods, creams, medications that could influence the performance of the tests, nor we considered the intentional search for infection by blastomycosis, coccidioidomycosis and paracoccidioidomycosis, the main agents that show cross-reaction with the *Histoplasma* antigen detection tests. |
| | **27** | Implications for practice, including the intended use and clinical role of the index test | Discussion; paragraph 14.<br>urine *Histoplasma* antigen detection tests showed excellent performance for the diagnosis of PDH in PLWHIV. The integration of these tests in clinical laboratories will certainly impact on early diagnosis and treatment, and consequently on the outcome of patients. These tests have an advantage over PCR tests, their functioning is higher, they are commercial standardized tests, easy and quick to perform, and provide results in minutes or hours. |
| **OTHER INFORMATION** | | | |
| | **28** | Registration number and name of registry | NA |
| | **29** | Where the full study protocol can be accessed | NA |
| | **30** | Sources of funding and other support; role of funders | Submission form |

# STARD 2015

## AIM

STARD stands for "Standards for Reporting Diagnostic accuracy studies". This list of items was developed to contribute to the completeness and transparency of reporting of diagnostic accuracy studies. Authors can use the list to write informative study reports. Editors and peer-reviewers can use it to evaluate whether the information has been included in manuscripts submitted for publication.

## EXPLANATION

A **diagnostic accuracy study** evaluates the ability of one or more medical tests to correctly classify study participants as having a **target condition.** This can be a disease, a disease stage, response or benefit from therapy, or an event or condition in the future. A medical test can be an imaging procedure, a laboratory test, elements from history and physical examination, a combination of these, or any other method for collecting information about the current health status of a patient.

The test whose accuracy is evaluated is called **index test.** A study can evaluate the accuracy of one or more index tests. Evaluating the ability of a medical test to correctly classify patients is typically done by comparing the distribution of the index test results with those of the **reference standard**. The reference standard is the best available method for establishing the presence or absence of the target condition. An accuracy study can rely on one or more reference standards.

If test results are categorized as either positive or negative, the cross tabulation of the index test results against those of the reference standard can be used to estimate the **sensitivity** of the index test (the proportion of participants *with* the target condition who have a positive index test), and its **specificity** (the proportion *without* the target condition who have a negative index test). From this cross tabulation (sometimes referred to as the contingency or "2x2" table), several other accuracy statistics can be estimated, such as the positive and negative **predictive values** of the test. Confidence intervals around estimates of accuracy can then be calculated to quantify the statistical **precision** of the measurements.

If the index test results can take more than two values, categorization of test results as positive or negative requires a **test positivity cut-off**. When multiple such cut-offs can be defined, authors can report a receiver operating characteristic (ROC) curve which graphically represents the combination of sensitivity and specificity for each possible test positivity cut-off. The **area under the ROC curve** informs in a single numerical value about the overall diagnostic accuracy of the index test.

The **intended use** of a medical test can be diagnosis, screening, staging, monitoring, surveillance, prediction or prognosis. The **clinical role** of a test explains its position relative to existing tests in the clinical pathway. A replacement test, for example, replaces an existing test. A triage test is used before an existing test; an add-on test is used after an existing test.

Besides diagnostic accuracy, several other outcomes and statistics may be relevant in the evaluation of medical tests. Medical tests can also be used to classify patients for purposes other than diagnosis, such as staging or prognosis. The STARD list was not explicitly developed for these other outcomes, statistics, and study types, although most STARD items would still apply.

## DEVELOPMENT

This STARD list was released in 2015. The 30 items were identified by an international expert group of methodologists, researchers, and editors. The guiding principle in the development of STARD was to select items that, when reported, would help readers to judge the potential for bias in the study, to appraise the applicability of the study findings and the validity of conclusions and recommendations. The list represents an update of the first version, which was published in 2003.

More information can be found on http://www.equator-network.org/reporting-guidelines/stard.
