# Supplementary material for: Diagnostic accuracy of antigen detection in urine and molecular assays testing in different clinical samples for the diagnosis of progressive disseminated histoplasmosis in patients living with HIV/AIDS: A prospective multicenter study in Mexico
Source: PLoS Negl Trop Dis. 2021 Mar 8;15(3):e0009215. doi: 10.1371/journal.pntd.0009215 (PMC7971897; doi:10.1371/journal.pntd.0009215)
Supplement: Attachment — Submitted filename: Review.pdf [file pntd.0009215.s002.pdf]

Diagnostic accuracy of antigen detection in urine and molecular assays testing in different clinical samples for the diagnosis of progressive disseminated histoplasmosis in patients living with HIV/AIDS: A prospective multicenter study in Mexico.

This is a well prepared manuscript. Data presented in this manuscript is valuable and will add important information for the improvement of histoplasmosis diagnosis and care.

I would like to highlight the good design of this study, and the way data was presented. This study evaluated a large number of patients, and lot of laboratory and clinical data. Information presented is clear and easy to follow and understand (congratulation to authors).

I think data presented here about molecular testing is also valuable.

Some aspect needs to be improved, in order to do this manuscript much better.

Minor:

1. Please check the use of italics in microorganism names, in special "*Histoplasma*".
2. Did you have any indeterminate results using the MiraVista LFA?
3. Do you have data of the *Histoplasma* EIA concentration? Can you present the median and rank of the concentration?
4. On Tables 1 and 2: it is possible to add data about co-infections and add CD4<100? Please also correct and extra comma after some of the values (age, HIV diagnosis, CD4, HIV VL…). Addtionally, in signs and symptoms, and X rays, I would like to suggest listed these from more frequent to less frequent.
5. Table 1: correct proportion of diarrhea (correct is 94.9%, or you can use 95.0%)

Major:

1. Please mention antigen test commercial names and products references. Please check products web-site.
    a. Clarus HISTOPLASMA GM ENZYME IMMUNOASSAY (https://www.immy.com/hgm)
    b. MiraVista Histoplasma urine antigen LFA (https://www.immy.com/hgm)
2. The IMMY ALPHA *Histoplasma* EIA is a product discontinued, this product was replaced by the Clarus HISTOPLASMA GM ENZYME IMMUNOASSAY (the IMMY ASR GM EIA your evaluated here). Do you think it is necessary to present this data? In special based in the fact that this data was previous published? (PLOS NTD Nov 5, 2018). I would like to suggest removed it, this could be help on shorter the final paper, and also help on avoid confusion on people not experts on histoplasmosis diagnosis.
3. Introduction: Please add more details in the introduction about the problem of the histoplasmosis and tuberculosis co-infection. Here some recommended references:
    a. Adenis et al. Am J Trop Med Hyg. 2014 Feb;90(2):216-23.
    b. Nacher et al. PLoS Negl Trop Dis. 2014 Dec 4;8(12):e3290.
    c. Adenis et al. Lancet Infect Dis. 2018 Oct;18(10):1150-1159.
    d. Caceres and Valdes. J Fungi (Basel). 2019 Aug 9;5(3):73.
4. Introduction: Please also mention in the introduction that the IMMY ASR GM EIA and the MiraVista lateral flow assay were previous validated, here the references:
    a. Caceres et al. Mycoses. 2020 Feb;63(2):139-144.
    b. Caceres et al. J Clin Microbiol. 2018 May 25;56(6):e01959-17.
5. Methods: please in "clinical definitions" add the EORCT/MSGREC reference.
6. Methods: please in "antigen detection procedures in urine samples" add more details of kits used (please see first major comment).

7. Methods: please check in "*Histoplasma* antigen detection in urine, using the IMMY ASR GM EIA" please check line 203 "The optical densities (OD) were read at 450 nm". Product package insert said on reading the test: "A dual wavelength reader is required, with absorbances read at 450 nm and 620/630 nm. Blank on the 1X Wash Buffer ( ). This assay has not been validated with a single wavelength reader". Additional, please correct in line 202; "0·4" by "0.4".
8. Results: this a great multicenter study, that add valuable information about histoplasmosis in Mexico (10 study places from seven Mexico states). I would like to suggest, add a map describing patient tested by region, and positivity by region. Please check figure 3 of this study published by Falci et al in Brazil (https://doi.org/10.1093/ofid/ofz073).
9. Results: authors did a great description of the 280 patients without histoplasmosis, in especial the description of co-infections. Is it same information available for the histoplasmosis cases? It is available, please added it. I am very curious in special for mycobacterial co-infections.
10. Results: Please, in table 3 add value of assay accuracy (this value is important to know correct proportion of classification).
11. Results: Line 411 and 424, please clarified that this false negative and positive results are from the antigen results.
12. Results: "false positive" On these 26 patients is available data of serology (immunodiffusion or complement Fixation)? What happened with data from molecular testing on these 26 patients? I think data from Hcp 100 nested PCR would be useful (not too sensitive, but specific).
13. Please improve figures resolution, I was not able to see information presented there (figures pending to review).
14. Discussion: Analytical performance of molecular test was not the best, but I consider these negative results add valuable information. Reading the discussion, I got the felling this data was ignored at the end of the manuscript. Please include some points like the detection of DNA in six of the seven false negatives of the antigen test. And also discuss the specificity issue with the 1281-1283220 SCAR nested PCR (compared with original validations results, Frias et at 2011).

As final comments, I hope the author with data of this study will prepare a second manuscript comparing patients with and without histoplasmosis.
