# Supplementary material for: Diagnostic accuracy of antigen detection in urine and molecular assays testing in different clinical samples for the diagnosis of progressive disseminated histoplasmosis in patients living with HIV/AIDS: A prospective multicenter study in Mexico
Source: PLoS Negl Trop Dis. 2021 Mar 8;15(3):e0009215. doi: 10.1371/journal.pntd.0009215 (PMC7971897; doi:10.1371/journal.pntd.0009215)
Supplement: Attachment — Submitted filename: Answer_to_Reviewers.docx [file pntd.0009215.s003.docx]

**Answers to the Reviewers:**

**Reviewer #1:**
    Some aspect needs to be improved, in order to do this manuscript much better.

**Minor:**

**Query 1:**  Please check the use of italics in microorganism names, in special “Histoplasma”.

**Answer:** It was reviewed and corrected in the manuscript.

**Query 2:** Did you have any indeterminate results using the MiraVista LFA?

**Answer:** No, we didn't have any indeterminate results using the MiraVista LFA.

**Query 3:** Do you have data of the *Histoplasma* EIA concentration? Can you present the median and rank of the concentration?

**Answer:** The range of antigen concentration in the cases detected as positive by clarus *Histoplasma* GM Enzyme Immunoassay was 0.43998 ng/mL – >25 ng/mL, and the median was >25 ng/mL. It was reviewed and added to the manuscript.

**Query 4:** On Tables 1 and 2: it is possible to add data about co-infections and add CD4<100?

**Answer:** Coinfection data in patients with proven PDH and probable PDH were added in Figure 1 and Table 5.

**Query 5:** Please also correct and extra comma after some of the values (age, HIV diagnosis, CD4, HIV VL…). Additionally, in signs and symptoms, and X rays, I would like to suggest listed these from more frequent to less frequent.

**Answer:** It was reviewed and corrected in the manuscript.

**Query 6:** Table 1: correct proportion of diarrhea (correct is 94.9%, or you can use 95.0%)

**Answer:** In relation to this point, we make the clarification that the real percentages are 50.9%, 58.5% and 47%.

**Major:**

**Query 1:** Please mention antigen test commercial names and products references. Please check products web-site.

a. Clarus HISTOPLASMA GM ENZYME IMMUNOASSAY (https://www.immy.com/hgm)
b. MiraVista Histoplasma urine antigen LFA (https://www.immy.com/hgm)

**Answer:** It was reviewed and corrected in the manuscript.

**Query 2:** The IMMY ALPHA *Histoplasma* EIA is a product discontinued, this product was replaced by the Clarus HISTOPLASMA GM ENZYME IMMUNOASSAY (the IMMY ASR GM EIA your evaluated here). Do you think it is necessary to present this data? In special based in the fact that this data was previous published? (PLOS NTD Nov 5, 2018). I would like to suggest removed it, this could be help on shorter the final paper, and also help on avoid confusion on people not experts on histoplasmosis diagnosis.

**Answer:** At the time of the development of this project, IMMY ALPHA Histoplasma EIA was in force; in addition, this article shows the results of a larger sample (391 patients who were studies with the three methods). These results clearly show the improvement of IMMY with the second generation ELISA type assay.

**Query 3:** Introduction: Please add more details in the introduction about the problem of the histoplasmosis and tuberculosis co-infection. Here some recommended references: a. Adenis et al. a. Am J Trop Med Hyg. 2014 Feb;90(2):216-23.

b. Nacher et al. PLoS Negl Trop Dis. 2014 Dec 4;8(12):e3290.
c. Adenis et al. Lancet Infect Dis. 2018 Oct;18(10):1150-1159.
d. Caceres and Valdes. J Fungi (Basel). 2019 Aug 9;5(3):73.

**Answer:** The references were reviewed, and the information was added to the manuscript.

**Query 4:** Introduction: Please also mention in the introduction that the IMMY ASR GM EIA and the MiraVista lateral flow assay were previous validated, here the references:

a. Caceres et al. Mycoses. 2020 Feb;63(2):139-144.
b. Caceres et al. J Clin Microbiol. 2018 May 25;56(6):e01959-17.

**Answer:** They have already been added to the manuscript.

**Query 5:** Methods: please in “clinical definitions” add the EORCT/MSGREC reference.
**Answer:** It has already been added to the text.

**Query 6:** Methods: please in “antigen detection procedures in urine samples” add more details of kits used (please see first major comment).

**Answer:** It has already been added to the manuscript.

**Query 7:**  Methods: please check in “Histoplasma antigen detection in urine, using the IMMY ASR GM EIA” please check line 203 “The optical densities (OD) were read at 450 nm”. Product package insert said on reading the test: “A dual wavelength reader is required, with absorbances read at 450 nm and 620/630 nm. Blank on the 1X Wash Buffer ( ). This assay has not been validated with a single wavelength reader”. Additional, please correct in line 202; “0·4” by “0.4”.

**Answer:** The reading was made at 450 nm and 620/630, therefore the description was corrected. Line 202 was corrected

**Query 8:** Results: this a great multicenter study, that add valuable information about histoplasmosis in Mexico (10 study places from seven Mexico states). I would like to suggest, add a map describing patient tested by region, and positivity by region. Please check figure 3 of this study published by Falci et al in Brazil (<https://doi.org/10.1093/ofid/ofz073>).

**Answer:** This observation seems interesting to us, however, we consider that adding a map contributes little to the objective of this publication, whose main goal is to emphasize the performance of histoplasmosis diagnostic methods; and also makes it more extensive.

Due to the importance of this knowledge, we are doing a clinical epidemiological analysis of histoplasmosis in this population, to present it in another document that we are preparing as a formal publication, in which we are including the previously mentioned map (the same that we have presented at INFOCUS 2018 in Colombia).

**Query 9:** Results: authors did a great description of the 280 patients without histoplasmosis, in especial the description of co-infections. Is it same information available for the histoplasmosis cases? It is available, please added it. I am very curious in special for mycobacterial co-infections.
**Answer:** We have added a detailed description of these data for the three important groups (proven PDH, probable PDH and group without histoplasmosis) in Table 1 and in Figure 1.

**Query 10:**  Results: Please, in table 3 add value of assay accuracy (this value is important to know correct proportion of classification).

**Answer:** The data was added to the table.

**Query 11:** Results: Line 411 and 424, please clarified that this false negative and positive results are from the antigen results.

**Answer:** It was cleared in the text.

**Query 12:** Results: “false positive” On these 26 patients is available data of serology (immunodiffusion or complement Fixation)?

**Answer:** No, we did not include serological tests in the study.

**Query 13:** What happened with data from molecular testing on these 26 patients? I think data from Hcp 100 nested PCR would be useful (not too sensitive, but specific).

**Answer:** Based on the observations made by the reviewer, we included, for the formal analysis, only 391 patients who had all three urine antigen detection tests. Thus, we defined a subgroup of 41 patients with a diagnosis of probable PDH according to the definitions of the EORTC / MSGREC. In it, we identified two more subgroups, a) 25 with probable PDH and without additional diagnosis and b) 16 more subjects with probable PDH and with an additional diagnosis.

The data from the molecular tests of these 41 cases were included in Tables 4 and 5

**Query 14:** Please improve figures resolution, I was not able to see information presented there (figures pending to review).

**Answer:** The figures were reviewed and resolution was improved.

**Query 15:** Discussion: Analytical performance of molecular test was not the best, but I consider these negative results add valuable information. Reading the discussion, I got the felling this data was ignored at the end of the manuscript. Please include some points like the detection of DNA in six of the seven false negatives of the antigen test.

**Answer:** In fact, Indeed, when we reviewed the results of the seven cases with negative Histoplasma antigen urine tests, we found that 2/7 had positive cultures, 5/7 showed positive results in histopathology. Regarding the molecular tests, the nested PCR Hcp100 showed positivity for 3/7 (both cases with positive culture), while 1281-1283220 SCAR showed positive results for 6/7. The sensitivity (18.1 - 71.4%) and specificity (40.4 - 90.4%) of this test are highly variable depending on the sample analyzed. For this reason, we do not consider molecular tests as a tool of choice for diagnostic definition in cases with suspected histoplasmosis and with negative results in the Histoplasma antigen detection test in urine. In any case, if the clinical suspicion is high, it would be convenient to start treatment pending the results of the cultures or histopathological studies, in the understanding that this scenario corresponds to 7/391 (1.8%) of the cases.

These false negative results are probably due to the existence of some inhibitor in the samples (since all three antigen detection tests were negative), however we do not know this information.

**Query 16:** And also discuss the specificity issue with the 1281-1283_220_ SCAR nested PCR (compared with original validations results, Frias et at 2011).

**Answer:** The comment on the work by Frias et al. of 2011 seems interesting to us, however, it is necessary to emphasize that in this study the description of the method and its initial analytical validation were carried out, not a field diagnostic validation. The specificity of the 1281-1283_220_ SCAR markers was determined by performing PCR from genomic DNA from *H. capsulatum* isolates and from *C. immitis, C. posadasii, P. brasiliensis, B. dermatitidis, A. fumigatus, A. niger, C. neoformans, C. albicans, S. schenckii, C. carmichaelii*, and *Malbranchea sp*. and *M. tuberculosis*).Therefore, we understand the high specificity found.

In our multicenter, prospective, double-blind cohort study of patients with HIV and suspected diagnosis of disseminated and progressive histoplasmosis, we are describing the real diagnostic performance of this molecular test. This may explain the differences in specificity found in both studies.

**Query 17:** As final comments, I hope the author with data of this study will prepare a second manuscript comparing patients with and without histoplasmosis.

**Answer:** A second manuscript is being prepared, in which the clinical and epidemiological data of the different populations included in the study (patients with and without histoplasmosis, patients with TB, and patients with *Histoplasma* + TB). In fact, we have decided to write a third manuscript regarding molecular testing for diagnosing histoplasmosis.

**Reviewer #2:**

**METHODS**
 **Query 1:** Ag detection procedures, sample preparation of molecular assays and molecular assay procedures could be pushed to an appendix to commit to the objective of reducing the word number.

**Answer:** We believe that it is convenient to keep the description of the methods in the same document where diagnostic tests are compared. However, we would understand if the reviewers and editorial committee would prefer it to be submitted as supplemental material.

**Query 2:**  Regarding Ag detection procedures, information regarding the number of wells per patients is missing and also what was the threshold considered regarding CV in order to decide if a new test is necessary or not.

**Answer:** One sample per patient was analyzed and only once. To validate the results, we follow the manufacturer's recommendations, which refer that, “an assay is considered valid when the standard curve, positive control, negative control, and R2 (Correlation Coefficient) fall within the acceptable ranges.

**Query 3:** Are there samples that were run several times for any reasons or not? This could give informations regarding quality control procedures performed or not on the different run.

**Answer:** A batch analyzed with the clarus Histoplasma GM Enzyme Immunoassay kit was repeated, since one of the control parameters (Standards) was outside the acceptable limits, so we don’t have enough information to develop a CV.

**Query 4:** Statistics are correct and adapted to the design. Still no information regarding the statistical power was given. But this can be approach using the confidence interval mentioned when necessary across the manuscript.

**Answer:** We have incorporated IC values in all areas where it applies.

**Query 5:** Ethics met probably the mexican regulations but not sure since the study was not registered at the national level.

**Answer:** The protocol was reviewed and approved by the Human Ethics and Biomedical Research Committees of each of the participating centers.

    --------------------

**RESULTS**

**Query 6:** There are several tables and figures. If all of them are useful it may be helpful to merge tables as recommended below.

**Answer:** According to this suggestion, the original tables 1 and 2 were merged into the current table 1.

   **Query 7:** Authors should merge Table 1 and 2 to save the number of tables and give a comprehensive overview of baseline data for the total population, the proven Progressive Disseminated Histoplasmosis (PDH) cases and add both the characteristics of probable PDH and patients without proven PDH.

**Answer:** Tables 1 and 2 were merged, and the characteristics of the three groups (proven PDH, probable PDH and without PDH) were added.

 **Query 8:** First column with the 415 patients, 2nd clumn with the 108 confirmed, 27 probable and 280 without PDH, raw data column results should express n/N and percentage in (). This table will enable harmonization of the raw data because informations was missed in between the two tables. Please correct typos in the tables, notably kommas duplicate or missing informations after kommas.

**Answer:** It was reviewed and corrected in the manuscript.

**Query 9:** If available in population characteristics, time from ART initiation would be of high relevance since about 1/2 patients included and similar for PDH proven are not ART naive, informing on the context of potential infectious IRIS when discovering/diagnosing the disease. The context of IRIS may be relevant when discussing the false positive and negative patients.

**Answer:** We agree that IRIS is an important condition to consider in PLWHIV and histoplasmosis, however, we do not have information on the start date of treatment. However, in the groups with false negative results and cases with probable PDH, we were able to collect information regarding the type of ART (see Tables 3, 4 and 5).

**Query 10:** As the information is probably available, case-fatality rate should be expressed in the four populations listed above.

**Answer:** The information was added to table 1.

**Query 11:** It is not clearly explained why the total number and the number of PDH-proven urine samples for antigen detection varies from one test to another.  Please clarify. It may help explain variation in agreement in between antigen detection tests. Another proposal, which may have a low impact on statistical power would be to only consider those samples that were available for all antigen detection methods.

**Answer:** Based on this observation and suggestion, the analysis was adjusted to the 391 cases that were tested by the three tests.

   **DISCUSSION
Query 12:**  An important reference is lacking. Authors should have mentionned the recently release WHO Guidelines on diagnosing and treating histoplasmosis in people living with HIV.

**Answer:** It was revised and added to the manuscript

    --------------------
**Summary and General Comments**

**Query 13:** When considering the required relevance of the discussion and simplification of the manuscript in general, it might have been better to submit experiments on antigen detection and PCR in two separate manuscripts.

**Answer:** We find this observation very interesting; however, we believe that it is a magnificent opportunity to present the performance of current diagnostic tools in the same document, which greatly helps the reader to understand the scope of each one of them.

--------------------
